# 3T DCE-MRI Radiomics Improves Predictive Models of Complete Response to Neoadjuvant Chemotherapy in Breast Cancer

**DOI:** 10.3389/fonc.2021.630780

**Published:** 2021-04-20

**Authors:** Stefania Montemezzi, Giulio Benetti, Maria Vittoria Bisighin, Lucia Camera, Chiara Zerbato, Francesca Caumo, Elena Fiorio, Sara Zanelli, Michele Zuffante, Carlo Cavedon

**Affiliations:** ^1^ Radiology Unit, Azienda Ospedaliera Universitaria Integrata, Verona, Italy; ^2^ Medical Physics Unit, Azienda Ospedaliera Universitaria Integrata, Verona, Italy; ^3^ Radiology Unit, Istituto Oncologico Veneto – IRCCS, Padova, Italy; ^4^ Pathology Unit, Azienda Ospedaliera Universitaria Integrata, Verona, Italy; ^5^ Nuclear Medicine Unit, Azienda Ospedaliera Universitaria Integrata, Verona, Italy

**Keywords:** MRI, breast cancer, radiomics, medical imaging, machine learning, neoadjuvant chemotherapy, DCE

## Abstract

**Objectives:**

To test whether 3T MRI radiomics of breast malignant lesions improves the performance of predictive models of complete response to neoadjuvant chemotherapy when added to other clinical, histological and radiological information.

**Methods:**

Women who consecutively had pre-neoadjuvant chemotherapy (NAC) 3T DCE-MRI between January 2016 and October 2019 were retrospectively included in the study. 18F-FDG PET-CT and histological information obtained through lesion biopsy were also available. All patients underwent surgery and specimens were analyzed. Subjects were divided between complete responders (Pinder class 1i or 1ii) and non-complete responders to NAC. Geometric, first order or textural (higher order) radiomic features were extracted from pre-NAC MRI and feature reduction was performed. Five radiomic features were added to other available information to build predictive models of complete response to NAC using three different classifiers (logistic regression, support vector machines regression and random forest) and exploring the whole set of possible feature selections.

**Results:**

The study population consisted of 20 complete responders and 40 non-complete responders. Models including MRI radiomic features consistently showed better performance compared to combinations of other clinical, histological and radiological information. The AUC (ROC analysis) of predictors that did not include radiomic features reached up to 0.89, while all three classifiers gave AUC higher than 0.90 with the inclusion of radiomic information (range: 0.91-0.98).

**Conclusions:**

Radiomic features extracted from 3T DCE-MRI consistently improved predictive models of complete response to neo-adjuvant chemotherapy. However, further investigation is necessary before this information can be used for clinical decision making.

## Introduction

Neoadjuvant chemotherapy (NAC) is administered in large operable or locally advanced breast cancers to enable shrinkage of the tumor and allow breast-conserving surgery to be performed (1). The ideal goal of NAC is pathologic complete response (pCR) (2). Prior assessment of patients as potential complete responders to NAC would be of great clinical significance. Personalized NAC is widely used to treat triple-negative and HER2+ subtypes of breast cancer; further improvement of outcome prediction would allow personalized treatment to be efficiently administered to a larger group of patients.

MRI has been used extensively to assess treatment response to NAC, often in a multi-parametric approach (3). However, the predictive power of MRI parameters was seldom reported to improve the information already provided by the status of hormonal receptors (4). PET-CT has been used occasionally to monitor the response to NAC in breast cancer, sometimes in combination with MRI (5–7).

Radiomics consists of large-scale image analysis and association of “features” to biological or clinical endpoints (8). MRI radiomics has been used to search for associations between quantitative metrics and response to NAC. Eun et al. recently published a study on the association between textural features and the pathologic complete response to neo-adjuvant chemotherapy in breast cancer, finding that texture analysis of T1-weighted MRI at mid-treatment was predictive of complete response (9).

The simultaneous use of all the available information, including hormonal receptor status, DCE-MRI, DWI, PET-CT and radiomics features extracted from MRI, might have the potential to improve the capability of predicting complete response to NAC.

Machine learning algorithms have been used in breast cancer imaging for early prediction of response to neoadjuvant chemotherapy (10, 11). For example, Tahmassebi et al. tested eight machine learning-based classifiers on several quantitative and qualitative MRI parameters, which however did not include radiomic features (12).

The aim of this study was to test whether 3T MRI radiomics of breast malignant lesions improve the performance of predictive models of complete response to neoadjuvant chemotherapy when added to hormonal receptor status, MRI multi-parametric information and PET-CT.

## Materials and Methods

### Study Population

This retrospective study involved women who consecutively had pre-NAC dynamic contrast-enhanced 3T MRI (DCE-MRI) and 18F-FDG PET-CT between January 2016 and October 2019. The study was approved by the institutional review board of the Hospital. Patients gave consent to processing of their anonymized data. Patient exclusion criteria were: age<18 years, history of previous breast surgery, contraindications to performing MRI and/or administration of intravenous contrast medium, history of previous chemo- and/or radio-therapy.

Before NAC administration, histopathological information related to the expression of Ki-67, ER (Estrogen receptor), PgR (Progesterone receptor) and HER2 (Human Epidermal growth factor Receptor 2) were obtained through lesion biopsy.

Chemotherapy consisted in the sequential administration of anthracyclines and cyclophosphamide every three weeks for four cycles followed by taxanes once a week for twelve weeks. NAC lasted about 6 months and the therapeutic regimes were: ACx4 + TXLx12 and ECx4 + TXLx12 associated with Trastuzumab for one year in case of HER2 positive cancer. Patients underwent conservative surgery or mastectomy and sentinel lymph node biopsy or axillary dissection. Specimens were analyzed and patients were divided between complete responders (Pinder class 1i or 1ii) and non-complete responders (13). The distribution of the studied cohort and information on the receptors’ expression is reported in **Table 1**.

**Table 1:** Clinical, histological and radiological characteristics of the patients as a function of pathologic response.

	Responders	Non-responders	All	p-value
**Number**	20	40	60	—
**Age (y)**	49.2 (±11.6)	52,8 (±12.2)	51.6 (±12.0)	0.273
**ADC (x10^-6 mm^2/s)**	842 (±270)	875 (±197)	864 (±222)	0,629
** PET SUV Max**	8.44 (±5.08)	6.79 (±5.51)	7.34 (±5.39)	0,257
** Ki67 (%)**	39.7 (±23.0)	20.0 (±11.3)	26.6 (±18.5)	0,0013
** PgR (%)**	12.3 (±23.9)	34.6 (±33.9)	27.2 (±37.5)	0,0046
** ER (%)**	34.0 (±40.5)	80.8 (±26.2)	65.2 (±38.4)	0,0001
**Grade**				
2	2	23	25	0,0006
3	18	17	35	
**HER2**				
Pos	11	35	46	0,0088
Neg	9	5	14	
**Shape**				
I	8	27	35	0,0182
O	6	11	17	
R	6	2	8	
**Margin**				
I	16	19	35	0,0254
S	4	21	25	
**IntEnh**				
E	14	33	47	0,3654
O	1	3	4	
RE	5	4	9	
**Curve**				
I	0	3	3	0,6565
II	3	6	9	
III	17	31	48	
**Type**				
MC	6	18	24	0,4543
MF	5	6	11	
U	9	16	25	

Data are presented either as mean ± sd or number of patients with relative percentage. Shape: (I)rregular/(O)val/(R)ound; Margin: (I)rregular/(S)piculated; IntEnh: H(e)terogeneous/H(o)mogeneous/(R)im (E)nhancement; Type: (M)ulti(c)entric/(M)ulti(f)ocal/(U)nifocal.

### Imaging Protocol

The temporal evolution of the lesions during NAC was monitored by 3T DCE MRI (Achieva, Philips Medical Systems, Cleveland, Ohio, USA). The imaging protocol included contrast-enhanced 3D dynamic acquisition (THRIVE), fat-saturated (SPAIR) (TE=2ms, TR=shortest) before and after intravenous injection of 0.2 mL/kg of gadobenate dimeglumine or 0.1 mL/kg of Gadoteridol (Bracco Imaging, Milan, Italy), followed by a 20 mL saline flush, with a temporal resolution of 90 s; axial echo-planar (EPI) single-shot diffusion weighted imaging (TR/TE=shortest) with b-value=0 s/mm^2^ and 800 s/mm^2^. MRI was available at three time-points of therapy, however only the scans performed before NAC were used in this study.

MRI and PET-CT were performed between 30 and 2 days before NAC start. For PET, patients were injected with 2.5 MBq/Kg of 18F-FDG and scanned an hour after the administration of the radiopharmaceutical, with a scan time set to 3 min/bed.

### Radiomic Features Extraction and Reduction

Lesions were contoured in the subtracted image (pre-contrast image subtracted to the second dynamic image) by means of a semi-automatic commercial tool (HealthMyne, Madison, Wisconsin, USA) and verified by two radiologists with 35 and 5 years of experience in breast imaging, respectively. The two radiologists also evaluated morphologic MRI parameters and performed apparent diffusion coefficient (ADC) as well as SUV_max_ measurements.

Radiomic features were extracted from the VOI in both the third dynamic and subtracted images through PyRadiomics v2.2.0 (14). This choice is due to the fact that using only a subtracted dataset might eliminate relevant information which is not linked to contrast enhancement (e.g., textural features of non-enhancing tissue). Following the Image Biomarker Standardization Initiative (IBSI) guidelines, MRI gray levels were normalized before feature extraction (average and standard deviation forced to 0 and 100, respectively) and a fixed bin count of 8. Voxels were resampled to 0.90 mm cubes through b-splines interpolation. Before resampling, the image in-plane spacing, out-plane spacing and aspect ratio were 0.87±0.03 mm, 0.94±0.07 mm and 0.93±0.08, respectively.

For both the third dynamic and the subtracted image, a total of 107 features were extracted: geometric or zero order (14), first order (15), higher order or textural (75) (16).

A preliminary feature reduction was performed by combining LASSO regression analysis (17), logistic generalized linear model (18) and leave-one-out cross validation (LOOCV) (15). According to common practice in LASSO regression, all the covariates were standardized by subtracting their mean and dividing by their standard deviation. The value of lambda minimizing the mean LOOCV deviance was identified by means of the *glmnet* package for *R* (19) and used to select the radiomic features included in the study.

### Selection of the Most Significant Covariates

The pool of data was composed of six continuous variables (age, apparent diffusion coefficient (ADC), PET-CT SUV_max_, Ki-67 expression, ER and PgR expression) and seven categorical variables (lesion grade, HER-2 expression, shape, type of margin, internal enhancement (IntEnh), type of contrast enhancement kinetic curve (I=persistently enhancing – II=plateau – III=rapid wash out), type of lesion). Shape, margin and IntEnh were classified following the BiRADS guidelines for MRI in breast cancer whereas Ki-67, ER, PgR and HER-2 were obtained from the histological biopsy.

The significance test for each covariate was performed by means of the Welch Two Sample t-test when the variables were continuous and the Fisher’s Exact Test when the distribution was categorical.

Correlations between continuous variables were tested by means of the Spearman correlation coefficient; for categorical variables with a limited number of events per class the Fisher’s Exact Test was used.

An exhaustive approach was adopted to select the most significant covariates, *i.e.* a different model was built for each possible subset of covariates. Three classifiers were trained and tested: Logistic Regression (Logit), linear Support Vector machines Regression (SVR) and Random Forest (RF). The optimal hyperparameters (cost for linear SVR and number of trees for RF) were obtained on the most general LOOCV model (with all the covariates) and held constant for the entire process.

The AUC of each classifier for each subset of variables was estimated by averaging the AUCs obtained in both the 60-fold (leave-one-out) and one 30-fold (leave-two-out) cross validated models. The use of two different cross-validation schemes increases the stability of the predicted performances. Confidence intervals were estimated by using the ci.auc function of the pROC package (v1.16.2). The significance of each AUC curve was estimated against the null hypothesis H0: AUC=0.5 through the Mason-Graham process (20) and all the obtained p-values were multiplicity-corrected to limit the false-discovery rates (21).

### Predictive Models

The variables were divided into five groups:

- Group_1 (*G1*={Age, ADC, SUV_max_, grade, shape, margin, IntEnh, type of curve, type of lesion}): variables obtained from clinical and radiological data, without radiomics or histological information
- Group_2 (*Rad*): radiomic features that passed the reduction process
- Group_3 (*Hist*={Ki-67, ER, PgR, HER-2}): histological information
- Group_4 (*NoRad*={*G1, Hist*}): whole dataset without radiomic features
- Group_5 (*All*={*G1, Rad, Hist*}): whole dataset including radiomic features

The predictive performance for the classification task for each one of the classes above was evaluated with the three classifiers (Logit, SVR, RF). The importance of each feature was assessed as the frequency with which the same feature was included in the six models with higher average AUC.

## Results

### Study Population

An overview of all the available clinical, histological and radiological information is shown in **Table 1**, where the rightmost column shows the p-values resulting from the t-test or Fischer’s exact test.

### Radiomic Features Extraction and Reduction

**Figure 1:**
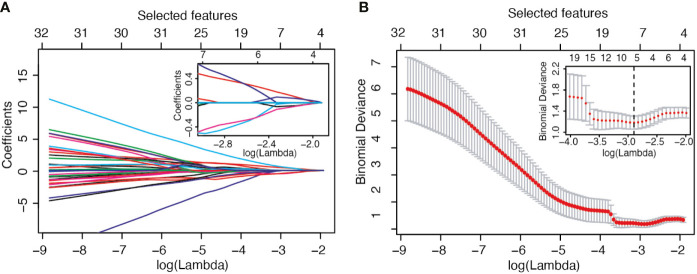
LASSO variable selection process. **(A)** Values of the LASSO regression coefficient as a function of log (Lambda). **(B)** LOOCV deviance as a function of log (Lambda) and therefore of the number of selected features.

The selection process operated by LASSO is represented in **Figure 1**. In panel B, the LOOCV deviance is plotted against λ; the value of lambda corresponding to the minimum deviance is identified in the inset by the dotted vertical line. From the whole set of 214 radiomic features, 5 passed the pre-selection process: Sphericity (F1), Kurtosis (F2), Dependence_Variance (F3), Long_Run_High_Gray_Level_Emphasis (F4), and High_Gray_Level_Zone_Emphasis (F5). Three of them (F2, F4, F5) were calculated on the second dynamic image after contrast medium injection while the remaining (F1, F3) were extracted from the subtracted image.

### Selection of the Most Significant Covariates and Predictive Models

**Figure 2:**
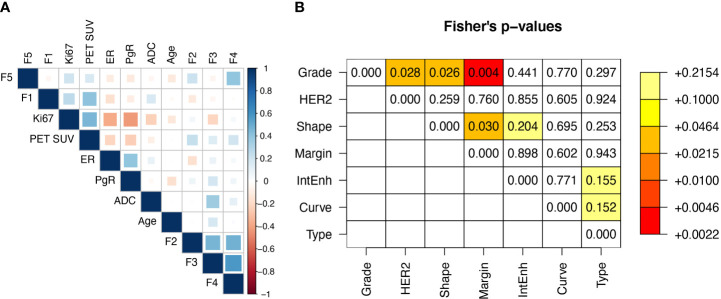
**(A)** Spearman correlation matrix between real variables and **(B)** Fisher’s p-values matrix between categorical variables.

Including the 5 radiomic features above, the total number of covariates was 18 (11 continuous and 7 categorical or dichotomous, see also **Table 1**). The correlation matrix between continuous variables is reported in **Figure 2A**. The highest correlation was observed between F3 and F4 (r=0.58), while all the other coefficients were below 0.5 (in absolute value). **Figure 2B** shows the Fisher’s exact test comparison between categorical variables. Low p-values are highlighted. The strongest correlation was observed between *Margin* and *Grade*. Overall, the continuous variables can be considered independent variables.

**Figure 3:**
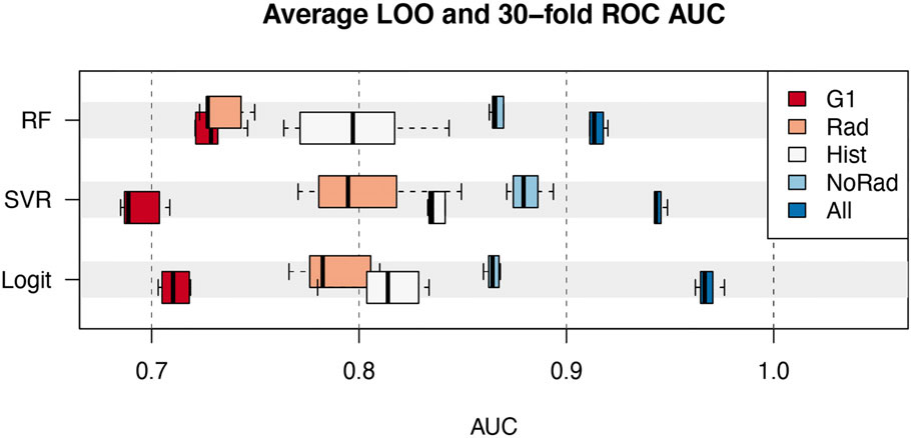
Average AUC of the best 6 models as a function of the class of variables (G1, Rad, Hist, NoRad and All) and classifier (RF, random forest; SVR, Support Vector machines Regression; Logit, Logistic regression). Boxplots represent the median value, interquartile range and extremes.

All the 2^18^ possible subsets of variables were tested in the selection process. The performance of the best 6 models for each group of variables is shown in **Figure 3**. The data represented by the boxplots are the average AUC in the ROC analysis of the 60-fold and 30-fold validations.

The models including the covariates in the *G1* group achieved a maximum AUC between 0.70 and 0.75. On the other hand, histological information (*Hist*) alone provided models with a maximum AUC spanning from 0.80 to 0.85. The combination of both *G1* and *Hist* classes pushed the performance between 0.85 and 0.90 for the three classifiers analyzed. The addition of the 5 radiomic features allowed AUC above 0.90 to be reached. In the latter case, the Logit model provided an AUC of 0.98 (CI=[0.94, 1.00]) for pCR~{F1+F2+F3+F4+Ki67+ER+Grade+HER2+Margin+Type} and 0.96 (CI=[0.92, 1.00]) for pCR~{F1+F2+F3+F4+ER+PgR+HER2+Margin+Type}. Overall, the average multiplicity-corrected p-value for the best 6 models in the All group was an order of magnitude lower than the others. For instance, $p_{All}^{best6,Logit}=4.44\;\times \;10-6$, $p_{NoRad}^{best6,Logit}=3.86\;\times \;10-4$ and $p_{Hist}^{best6,Logit}=1.74\;\times \;10-4$. The complete list of the average values of p for the best 6 models is visible in **Supplementary Table 1**.

**Figure 4:**
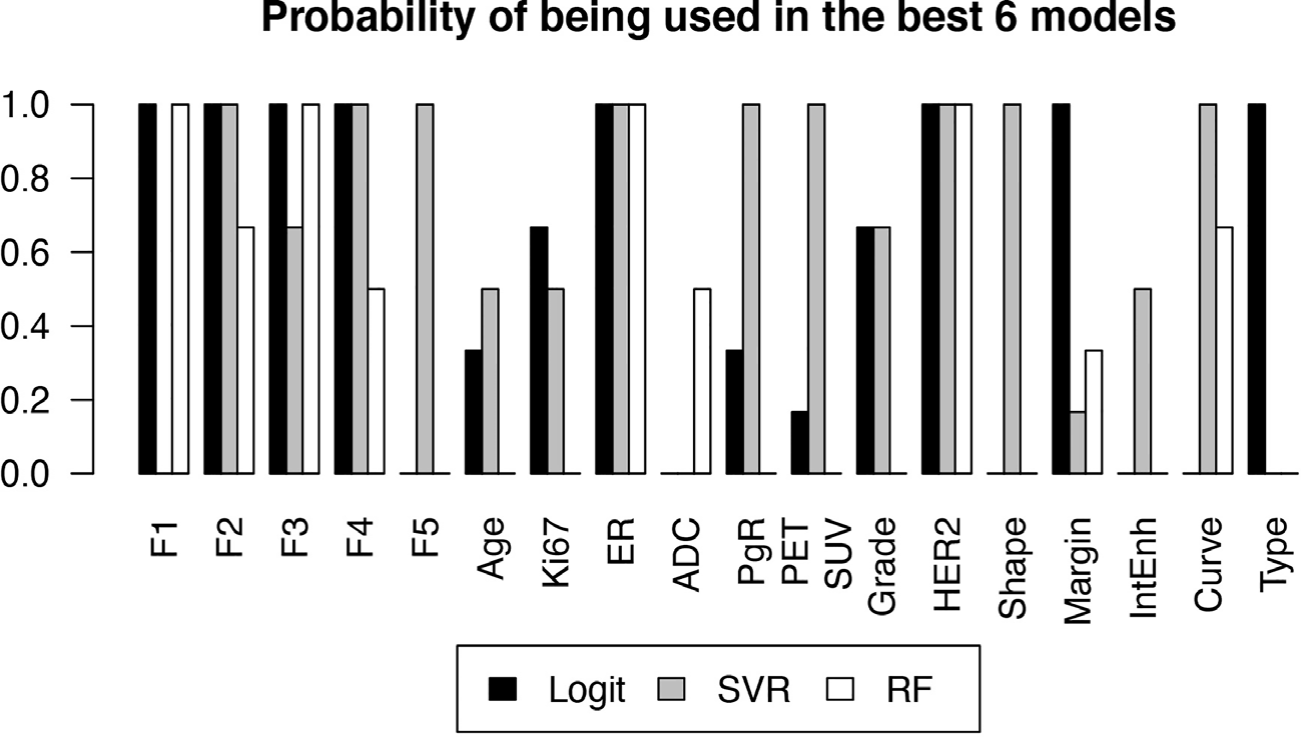
Probability of each variable of being included in a high-performance model, estimated by the frequency with which the variable was selected in one of the 6 best models, as a function of the classifier.

**Figure 4** reports the selection frequency of the included variables in the 6 models with the best performance, for each classifier.

The logistic regression model allows to see if the correlation of a covariate with pCR is positive or negative. The normalized regression coefficient m_j_ for covariate j has been computed as its weighted mean over the 2^18^ tested models:

$$m_{j}\equiv \sum_{i}\frac{m_{i,j}}{\sigma_{i,j}^{2}}/\sum_{i}\frac{1}{\sigma_{i,j}}$$

where m_i,j_ and σ_i,j_ are the i-th fit regression coefficient and error, respectively. The resulting values are reported in **Table 2**. Values in the interval [-1,1] indicate a non-significant average covariate correlation with pCR, whereas values higher than 1 or lower than -1 represent statistically relevant positive and negative correlations, respectively. As expected from the low p-values in **Table 1** and the high frequencies in **Figure 4**, the most correlated covariates were F2, F3, F4, Ki67, ER, HER2 and irregular shape. In this case, the categorical covariates have been converted in their dummy-variable counterparts to allow explicit definition of the regression coefficient.

**Table 2:** Regression correlation coefficients of the single covariates in the Logit model.

F1	F2	F3	F4	F5	Age	Ki67
0,9	1,8	-1,5	-2,0	0,8	-0,7	1,9
						
**ER**	**ADC**	**PgR**	**PET_SUV**	**Grade_3**	**HER2**	**Shape_I**
-2,5	0,2	-0,7	-0,6	1,4	2,2	-1,5
						
**Shape_O**	**Margin_I**	**IntEnh_E**	**IntEnh_O**	**Curve_III**	**Type_MC**	**Type_MF**
-1,2	1,0	-0,3	-0,2	0,3	0,1	0,6

Values higher than 1 and lower than -1 represent statistically relevant positive and negative correlation of predictors, respectively.

## Discussion

The relevance of clinical, histological and radiological information has been evaluated by means of the analysis made on different groups of variables. The introduction of MRI radiomic features showed the potential to significantly improve predictive models of pathologic complete response to neoadjuvant chemotherapy, as can be seen in fig. 3. This result stands for the robustness of the additional information provided by radiomic features, confirmed by the consistent results obtained through multiple combination of variables. From the clinical standpoint, this is important as models that include radiomic information may offer a better prediction of complete response to therapy compared to protocols based on the hormonal receptor status alone.

These results compare to those of Chamming’s et al. (22), who obtained an AUC of 0.834 in ROC analysis with a model based on logistic regression and the inclusion of kurtosis, one of the radiomic features that resulted significant in our study. Eun et al. (9) used a model based on random forest on 3T-MRI texture analysis, after comparison with other 6 machine-learning based classifiers, and obtained an AUC of 0.82 using metrics calculated on the mid-treatment contrast-enhanced T1 MRI. Fan et al. (23) found results comparable to the outcome of this study, with an AUC reaching up to 0.91 using a subset of 12 radiomic features selected among 158 metrics for prediction of pathologic complete response to NAC. Different from our approach, they included signatures extracted from the background parenchyma in the predictive model; however, they did not include other MRI and PET-CT information, nor histological characteristics of the lesions. Bian et al. (24) analyzed a pool of 152 patients and found a potential predictive power of T2W MRI radiomic metrics for NAC treatment outcome. Differently from their study, our investigation combines multiple information including PET-CT metrics and hormonal receptor status, in an attempt to maximize the predictive performance of the models. Furthermore, though based on a smaller number of patients, our study included a strong feature reduction strategy in order to reduce overfitting, with final models based on five out of the total 214 initial radiomic features compared to 18-20 out of more than 7000 features in the cited investigation. Sutton et al. (25) performed an extensive investigation on 273 patients, showing that MRI radiomic features combined to information on the molecular subtype allows an accurate classification of pCR, an approach similar to our study. That work, however, did not include other MRI parameters such as ADC or PET-CT metrics; furthermore, it was based on mixed 1.5T and 3.0T MRI. Zhou et al. (26) published a study on 55 patients based on 3.0T MRI only, showing the potential of radiomic features to predict NAC outcome; however, similarly to several other studies, they did not include additional non-radiomics and non-MRI-based information in their models.

Radiomic signatures that passed the feature selection process included sphericity and kurtosis. The latter was found to be a significant covariate also by Chamming’s et al. In the logit model, sphericity was positively correlated to pCR. This may be explained by the tendency of triple negative tumors – that generally respond better to NAC compared to other molecular subtypes – to present with round or oval shapes (27). The other three radiomic features used in the models are higher-order metrics associated with micro-inhomogeneities within the tumor.

From the results reported in figure 4 it can be seen that ER and HER2 are always contained in the best-performing models, due to their strong predictive power also shown by the low p-values in **Table 1**. This was expected as the estrogen and HER2 status are well known predictors of the response to therapy (28). Their negative (ER) and positive (HER2) correlations to pCR in the logit model, visible in **Table 2**, are also consistent with current knowledge.

F2, F3 and F4 were also consistently observed in the models that showed high predictive power. F2 corresponds to the kurtosis, already observed to be correlated to pCR (23). **Table 2** shows that the observed correlation in the logit model was positive, meaning that higher kurtosis correlates to better outcome. F3 and F4 are the Dependence Variance and Long Run High Gray Level Emphasis, respectively, and their negative correlation to pCR (**Table 2**) does not have an obvious interpretation, though their connection to tissue heterogeneity might suggest a characterization of the tumor microenvironment compared to the surrounding parenchyma.

This study has limitations. Firstly, it was a single-institution study, and the number of complete responders was limited. Furthermore, the retrospective nature of the investigation might have caused selection bias. Tumor segmentation was standardized by the use of a semi-automatic tool, however two radiologists reviewed and, in some instances, had to modify lesion contours in order to adjust initial mismatch. Finally, radiomic features were extracted from visible lesions only, while the surrounding parenchyma, possibly offering additional hidden information, was not included in the analysis.

In conclusion, radiomic features extracted from pre-NAC contrast-enhanced 3T MRI consistently improved the performance of predictive models when added to other clinical, histological and radiological data. However, further investigation is necessary before this information can be used for clinical decision making, especially due to the limited cases/variable ratio (6 for the model with 10 variables). If validated on a larger, independent, multi-institutional study, this analysis may become an important tool for predicting response to NAC for breast cancer.

## Data Availability Statement

The raw data supporting the conclusions of this article will be made available by the authors, without undue reservation.

## Ethics Statement

This study was approved by the institutional review board of the Hospital. Patients gave consent to processing of their anonymized data.

## Author Contributions

Conceptualization, SM, GB and CC. Data curation and statistical analysis, GB and CC. Writing—original draft preparation, SM, GB, MZ and CC. Writing—review and editing, SM, GB, FC, MB, LC, CZ, EF, SZ, MZ and CC. All authors contributed to the article and approved the submitted version.

## Conflict of Interest

The authors declare that the research was conducted in the absence of any commercial or financial relationships that could be construed as a potential conflict of interest.
