# Supplementary material for: 3T DCE-MRI Radiomics Improves Predictive Models of Complete Response to Neoadjuvant Chemotherapy in Breast Cancer
Source: Front Oncol. 2021 Apr 20;11:630780. doi: 10.3389/fonc.2021.630780 (PMC8093630; doi:10.3389/fonc.2021.630780)
Supplement: Supplementary file 1 [file DataSheet_1.pdf]

Supplementary Material

1 Significance tests

p^{best6}	Logit	SVR	RF		p^{best6} / p^{best6}_{All}	Logit	SVR	RF
**G1**	1,33 E-1	4,65 E-1	9,64 E-2		**G1**	29983	165069	1651
**Rad**	1,01 E-3	6,32 E-4	1,81 E-2		**Rad**	228	224	310
**Hist**	1,74 E-4	2,59 E-5	5,18 E-4		**Hist**	39	9	9
**NoRad**	3,86 E-4	4,99 E-5	5,29 E-4		**NoRad**	87	18	9
**All**	4,44 E-6	2,82 E-6	5,84 E-5		**All**	1	1	1

Supplementary Table 1. Average significance (p-values) of the best 6 models for each classifier type (Logistic regression, Support Vector Machines, Random Forest) and each group of covariates. P-values have been corrected for test multiplicity to limit the false discovery rate (Benjamini-Hochberg method, see main text) with the total number of elements of the covariates group. To ease the comparison the same values have been normalized to the *All* group, on the right side of the table (p^{best6} / p^{best6}_{All}).

2 AUC vs number of variables

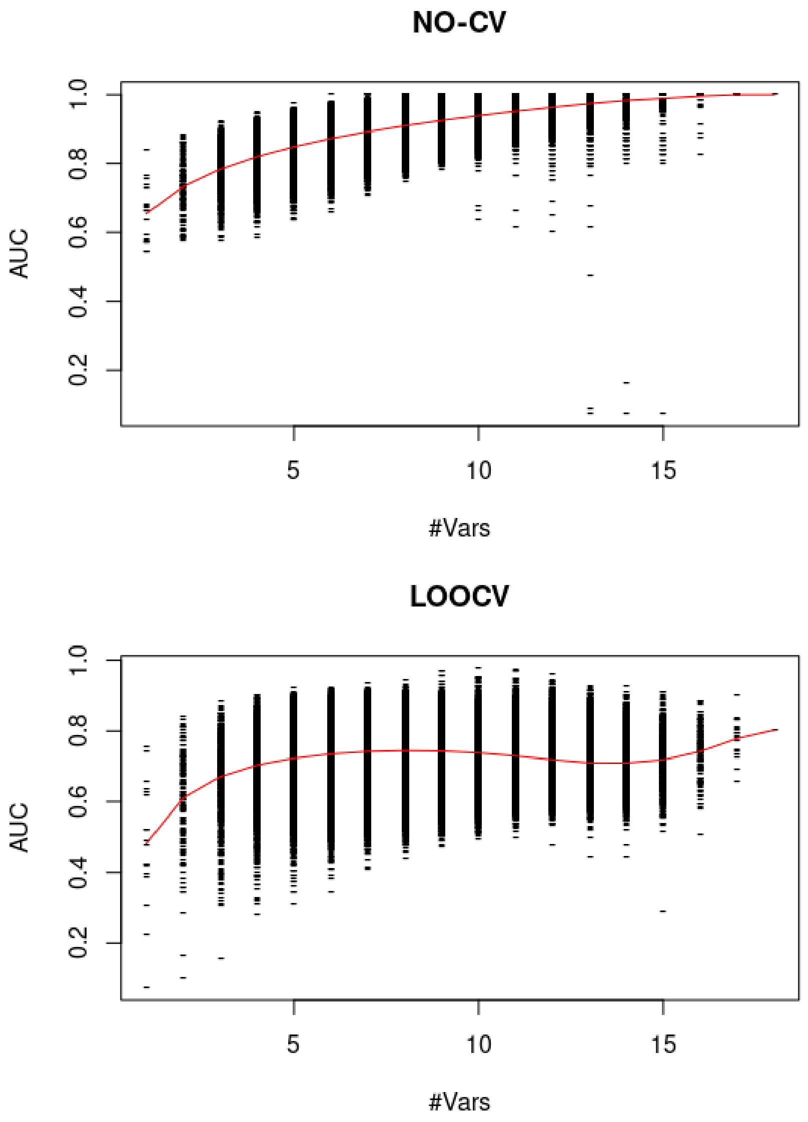

Supplementary Figure 1. AUC as a function of the number of used variables in all the studied models (about 280’000) for the non cross-validated (NO-CV, left) and the cross validated models (LOOCV, right). The red line is the average of all the AUC as a function of the number of used variables. Only one model contains all the variables (#Var = 18), visible as the rightmost dot in the graphs. Differently from the non cross-validated models, which tend to overfit when the number of variables is high, the CV ones have constant average AUCs up to 18 variables. Overfit in CV models provide a low AUC. Please note that the red line is not representative for the average performances of a well-defined model but the average among all the possible models built with this set of variables (and therefore also models omitting important information).

3 Probabilistic misclassification of the best 6 models.

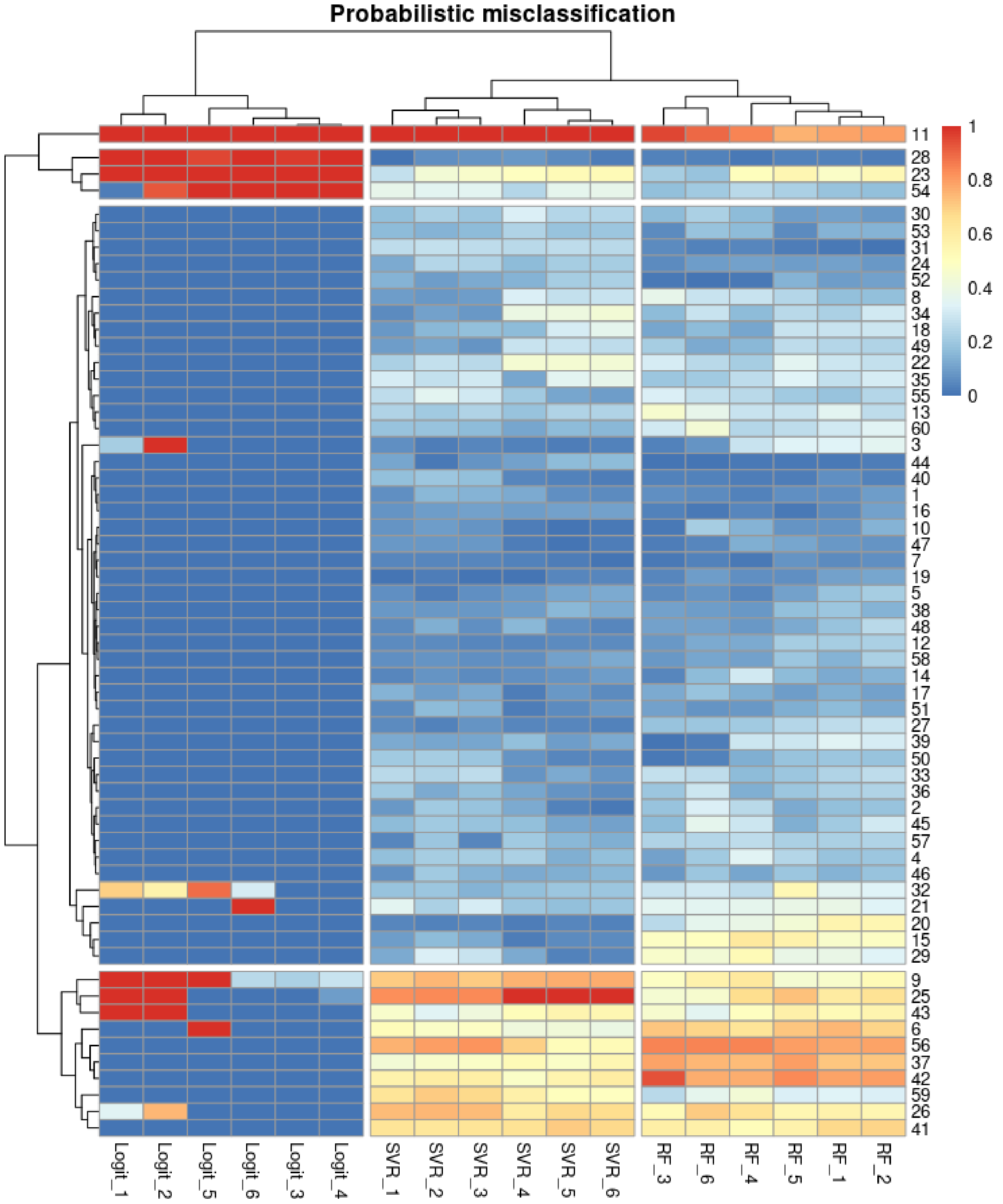

Supplementary Figure 2. Probabilistic Misclassification (colorbar) for each patient (rows) and model (columns). Probabilistic Misclassification (PM) is defined as |ti-pi| where pi is the predicted probability from the model for the case i and ti is the true value for the same case (either 0 or 1).
